# Chemical painful post-traumatic trigeminal neuropathy induced by dental bleaching: A case report

**DOI:** 10.4317/jced.60744

**Published:** 2024-01-01

**Authors:** Ashley Lebel, Tal Wizman, Géraldine Lescaille, Sandrine Dahan, Yves Boucher, Julia Bosco

**Affiliations:** 1 Service Odontologie, Hôpital Pitié Salpêtrière, Paris, France; 2 UFR Odontologie, Université Paris Cité, France; 3 Private practice; 4 Laboratoire de Neurobiologie Orofaciale (EA7543), Université Paris Cité, France

## Abstract

**Background:**

Teeth whitening is a frequent request in clinical practice. The most widely used whitening agent on vital teeth is carbamide peroxide. This article reports a rare adverse effect following a whitening procedure.

**Case description:**

A 29-year-old patient was referred to the dental emergency department for severe pain that exhibited the characteristics of neuropathic pain. In the absence of any visible lesion or traumatic event, this pain was linked to the recent application of carbamide peroxide (10%) during a bleaching procedure. The diagnosis of painful post-traumatic trigeminal neuropathy (PTTN) of chemical origin was made. Treatment with the anticonvulsant gabapentin (900mg per day) gradually reduced the pain until its complete disappearance. After presenting the clinical characteristics of the case, the pathophysiological hypotheses are discussed.

**Practical implications:**

Carbamide peroxide application may elicit nerve damage through a cascade of cellular and biological reactions, resulting in neuropathic pain. The successful management of this clinical case may provide useful information for similar situations.

** Key words:**Case report, carbamide peroxide, painful neuropathy, pain, gingiva.

## Introduction

At-home teeth whitening using a chemical agent applied in thermoformed trays is a routine technique. Carbamide peroxide is the most commonly used bleaching agent for vital teeth. It is a crystalline solid with the chemical formula CH6N2O3, composed of hydrogen peroxide stabilized in a solution of glycerin and coupled to a urea molecule (1). In contact with saliva and under the effect of oral temperature, it dissociates into urea and hydrogen peroxide, eventually resulting in the formation of free radicals (ROS) responsible for the bleaching effects. Because of possible adverse effects on tissues, both the concentration of the agent and the time of exposure must be controlled. Although it is usually well tolerated, this procedure may result in side effects such as dentin hypersensitivity or mechanical allodynia, reported by 15 to 78% of the patients (2). These adverse side effects are typically moderate and reversible and disappear after stopping the application. However, pain may persist in some cases, despite treatment discontinuation. This article reports the case of a patient who experienced several months of pain after a bleaching procedure. To the best of our knowledge, no other case has been reported in the literature.

## Case Report

This article has been written according to the CARE recommendations for clinical cases. Written consent has been obtained from the patient for the publication of her data.

**Figure 1:**
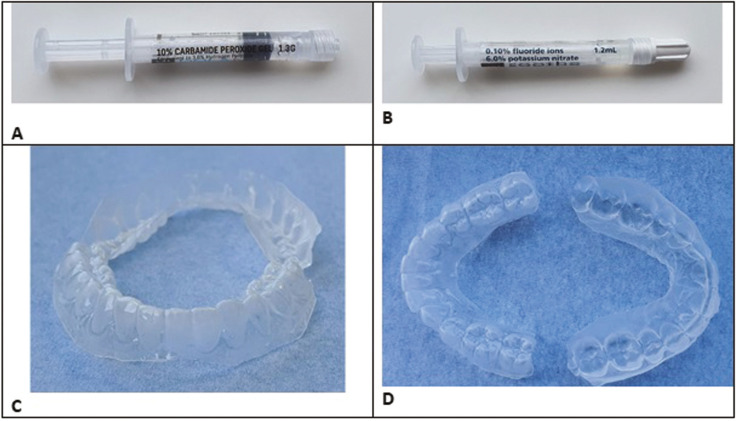
A. Whitening gel provided by the dentist for self-administration. B. gel provided by the dentist for relieving the pain elicited by the bleaching procedure. C. Thermoformed trays provided by the dentist for the application of the bleaching agent.

- Medical and dental history:

A 29-year-old woman, in good general health, was referred to the dental department of the hospital on 06/08/2021, because of intense pain following a dental whitening procedure. She declared no medical or surgical history. On 05/05/2021, she started a whitening procedure, provided by her dentist, consisting of the daily application of 10% carbamide peroxide gel (Fig. 1A) in thermoformed trays (Fig. 1C,D.). After two 2-hour applications, the patient experienced intense mandibular pain and interrupted treatment. She consulted her dentist who prescribed Elugel® (a water-based gel containing glycerin, sorbitol, hydroxymethyl cellulose, benzyl alcohol, carmine (CI 75470), chlorhexidine digluconate, aroma, glucose, limonene, sodium hydroxide) and a gel containing 0.1 % fluoride ions and 6 % potassium nitrate (Fig. 1B), without any noticeable improvement. The patient consulted her general physician on 06/04/2021, who referred her to the dental service of the GHPS and placed her on sick leave from work.

-Pain Consultation

**Figure 2:**
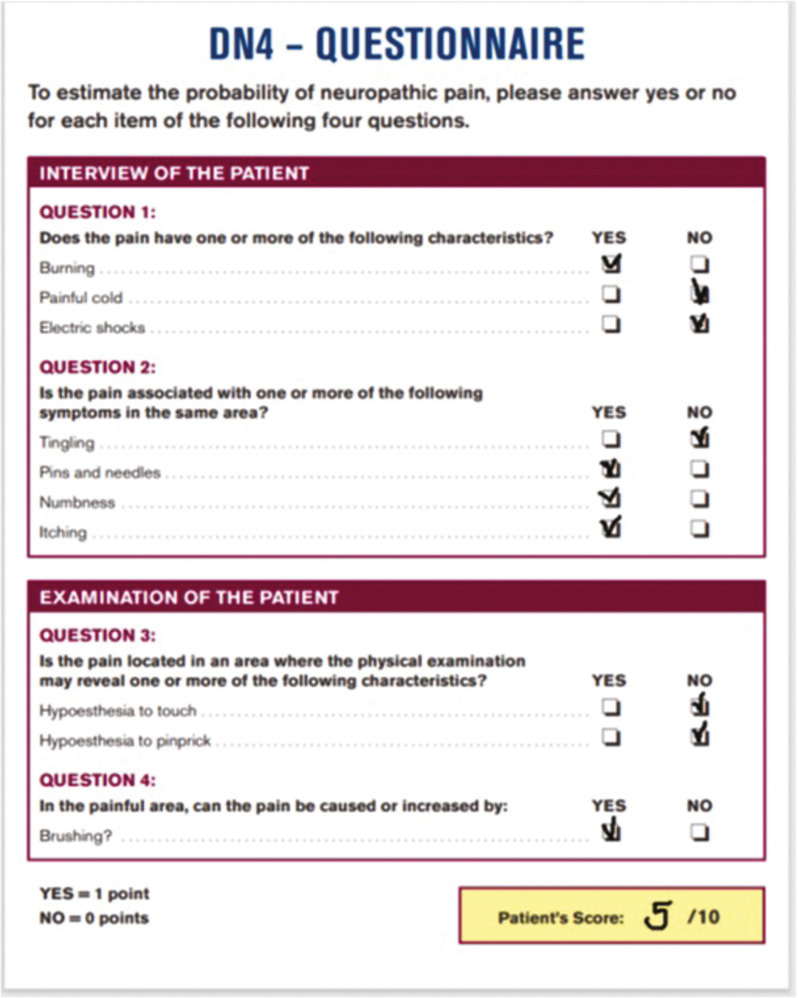
Translated DN4 questionnaire filled at the pain consultation. The total score was 5/10. The DN4 is used as a diagnostic tool and the cut-off for neuropathic pain is 4 (3).

On 06/28/2021, the pain consultation was conducted according to the SOCRATES recommendations (Site Onset Character Radiation Associated features Time course Exacerbating and relieving factors Severity). Pain started a few hours after the end of the second application of the bleaching gel and persisted thereafter. On the day of the appointment, the pain followed the same pattern throughout the day: discomfort upon awakening, evolving into pain, and culminating in the evening. The pain was strictly localized in the gingival area of teeth 33-43 and the lower lip. Pain quality (assessed using the DN4 questionnaire for neuropathic pain)(3) was mainly described as burning, numbness, pins and needles, and itching. The DN4 questionnaire score was 5/10 (Fig. 2). The patient reported dysesthesia when touching her teeth with the tongue. The mean intensity of pain was estimated at 5 on a numerical scale (0-10) with peaks estimated at 7/10. The pain was globally relieved during meals, but specific food such as acidic fruits and spices increased the pain. She did not report dysgeusia. Pain was not relieved with NSAIDs, acetaminophen, or codeine.

-Clinical examination

**Figure 3:**
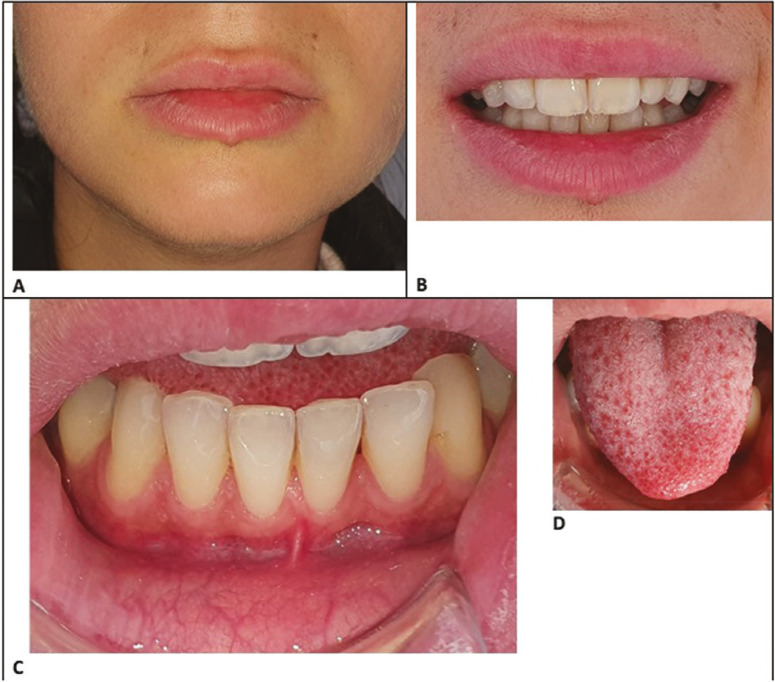
Photographic views of extra and intraoral tissues.

The patient presented good oral hygiene. Clinical examination (Fig. 3) revealed no cutaneous, mucosal, dental, or periodontal lesion. The patient reported mechanical allodynia in the gingival area of teeth 33-43. Pulp sensitivity (thermal and electrical) and mechanical tests were performed, with physiological responses in all teeth. Upon examination, the trays given by the dentist (Fig. 1C,D.) covered the gingival margins and therefore allowed the spreading of the bleaching gel on the soft tissues.

-Complementary examination 

**Figure 4:**
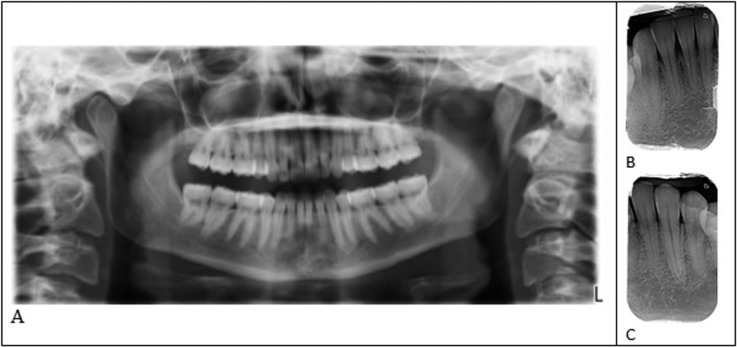
A. Panoramic radiography. B. Retroalveolar radiography teeth 32-43. C. Retroalveolar radiography, teeth 32-34.

Retroalveolar and panoramic X-rays revealed no dental or periodontal lesion that could explain the pain (Fig. 4).

-Diagnosis

Burning mouth syndrome, idiopathic neuropathy, and persistent orofacial idiopathic pain were considered differential diagnoses but discarded. Based on medical history, clinical examination, and images, the diagnosis of chemical-based painful post-traumatic trigeminal neuropathy was made, according to diagnostic criteria of the ICHD3 classification (4). As the patient met 4 of the 5 diagnostic criteria (A, B, C, and E), the probability of a neuropathic origin was high, according to the neuropathic diagnosis classification system.

-Treatment

Topical applications of Xylocaine (2%), Methylene blue (0.5%), and systemic Pregabalin (25mg, 3 times a day for 3 months, in titration) were prescribed.

-Follow-Up.

Follow-up was first achieved by phone calls. On 05/07/2021, the patient reported a small pain improvement. She stopped pregabalin because of undesirable effects (agitation, dizziness). Pregabalin was replaced by gabapentin (300mg, 3 times a day) in titration for 3 months). On 09/07/2021, the patient was unable to cope with the situation and went to the Medical Emergency Room. The CT brain revealed no abnormality. The physician first diagnosed an anxiety crisis, then evoked inflammatory pain, which confused the patient who was previously told that the pain was of neuropathic origin. The patient left without specific medication or treatment. On 07/11/2021, the patient was seen at the GHPS, accompanied by her mother, who revealed that her daughter had consulted the emergency room several years ago for an anxiety crisis, although the patient did not seem to recall the episode. The patient self-declared anxiety and kept focusing on the pain despite the treatment, complaining of their inefficiency and the number of appointments without any results. She feared experiencing pain throughout her life and showed an emotional amplification of her pain. A careful explanation of the sensory and emotional mechanisms of pain was given to her in simple words to decrease stress. The patient was referred to a sophrologist to learn basic emotional control through relaxation, breathing, and positive thinking. When she was recalled on 07/16/2021, the patient reported a small decrease in pain, evaluated at 4/10 for the daily average intensity on the NRS. On 07/20/2021, the mother called to report that her daughter refused to continue gabapentin, fearing side effects. The patient was examined at GHPS again, the day after. Additional explanations were given and the prescription of gabapentin was confirmed. The patient did not answer phone calls for three months (she had left for vacation). On 11/08/2021, she came to the follow-up visit smiling and pain-free. She declared having complied with all advice and treatments (sophrology, gabapentin). A progressive decrease of the gabapentin was then elicited. The patient missed the next appointments, which resulted in stopping the medication. She consulted again on 01/07/2022. Pain had resumed with an average daily intensity of 3/10 and the patient self-reported a small level of anxiety. Gabapentin was reinstated in titration up to 900mg/day and she went to additional sophrology sessions. The patient resumed work. On 04/12/2022, at the follow-up visit the patient reported no pain with Gabapentin 900mg/day. A progressive decrease of the gabapentin was decided and stopped completely in July 2022. At the follow-up visit on 11/14/2022, she reported a complete disappearance of pain. In January 2023 she was still pain-free, without medication.

## Discussion

This article reports a rare case of chemically-induced PTTN, successfully treated with gabapentin and cognitive/psychologic support. Although included in the ICHD3 classification (4), clinical descriptions of chemically induced PTTN are sparse in the medical and dental literature, hence the importance of clinical documentation.

-Clinical considerations

This case fulfilled diagnostic criteria for PTTN (4), with a rapid onset as for most PTTN instead of PPTN occurring after physical injury (such as post-endodontic treatment or dental extraction). Treatment of PTTN is difficult since there are no clear specific recommendations and the treatment is usually adapted from spinal painful neuropathies. In this case, capsaicin-induced desensitisation was not attempted, considering the risk of exacerbation, especially in this case where a chemically-induced alteration was suspected. We followed international recommendations that favor gabapentin and pregabalin in first intention with a preference for pregabalin for its anxiolytic effects. We also added a topical application of methylene blue for its electrochemical properties and possible therapeutic effect in this specific case (5). However, no clear improvement could be attributed to methylene blue since no decrease in pain occurred. Only when gabapentin was effectively taken did the situation improve, in association with psychological intervention (sophrology). In addition, the interruption of treatment resulted in a reappearance of the pain, validating the therapeutic effect of the drug.

-Pathophysiological considerations

Carbamide peroxide can induce undesirable effects on dental tissues (such as changes in enamel properties and composition (6) or reversible inflammation of the dental pulp (7) and soft tissue irritation (burning, gingival irritation) (8). Inflammatory reactions are likely mediated by the release of neuropeptides, since the peroxide dissociation reaction causes an increase in the extracellular concentration of the neuropeptide substance *P* (SP), which is involved in neuroinflammation and pain via the axon reflex. SP induces immune cell attraction, modulates inflammatory mediators’ release, and decreases sensory thresholds. A study conducted on healthy dental pulps showed an increase in SP expression after different bleaching procedures (9).

This clinical case suggests that carbamide peroxide may also induce adverse effects in nervous tissue that is susceptible to promote the development of neuropathic pain through free radicals during reactions of oxidative stress and the TRPA1 receptor (transient receptor potential ankyrin). TRPA1, which elicits a burning sensation when activated by irritant compounds such as mustard oil, is involved in inflammatory and neuropathic pain and mediates chemesthesis at the oral level (10). TRPA1 is considered to be a major oxidant sensor since many endogenous compounds linked to oxidative stress, including hydrogen peroxide, activate the channel (11). Under inflammatory conditions or tissue damage, ROS are formed by cells and damage lipids, proteins, and DNA. They activate TRPA1 expressed by neurons, which induces the release of SP and CGRP associated with neurogenic inflammation and a prolonged mechanical and thermal hypersensitivity state. More specifically, Chen *et al*. (12) observed in pulp stem cells treated with a 15% hydrogen peroxide gel for 90 min or 40% for 45 min inducing oxidative stress and ROS production, an increase in the expression of TRPA1, intracellular absorption of Ca2+, activation of PANX1 favoring the secretion of ATP and its action as a neurotransmitter. In addition, TRPA1 upregulated TNF-ß and IL-6, inducing inflammation. Oxidative stress altered the viability of pulp cells, leading to cytotoxicity and changes in pain processing mechanisms following the increase in TRPA1 (13). On the contrary, inhibition of TRPA1 and oxidative stress decreases pain behavior and mechanical and thermal hypersensitivity.

Other downstream effectors can be involved, such as TRPM2, a non-selective cation channel activated by hydrogen peroxide, highly expressed in different nervous tissues and associated with several neurological disorders, including neuropathic pain (14). Receptor activation by oxidative stress induces aberrant intracellular calcium accumulation, cell death of various cell types including neurons (14), neutrophil and macrophage chemotaxis at the site of nerve injury, and central sensitization. In TRPM2-knocked-out animals, neuropathic pain was attenuated and TRPM2 was involved in transitioning from acute to chronic pain (15).

## Conclusions

The clinical case presented illustrates the potential danger of carbamide peroxide and encourages the proper handling of bleaching products. Oxidative stress, generated by dissociating carbamide peroxide into hydrogen peroxide, can lead to inflammatory pain via the release of neuropeptides and nerve damage that can lead to PTTN. TRPA1 and TRPM2 receptors are candidates for mediating these effects at the cellular level.
